# An improved method for intracellular DNA (iDNA) recovery from terrestrial environments

**DOI:** 10.1002/mbo3.1369

**Published:** 2023-06-25

**Authors:** Diego Medina Caro, Lucas Horstmann, Lars Ganzert, Romulo Oses, Thomas Friedl, Dirk Wagner

**Affiliations:** ^1^ GFZ German Research Centre for Geosciences Section Geomicrobiology Potsdam Germany; ^2^ Department Experimental Phycology and Culture Collection of Algae (EPSAG), Albrecht‐von‐Haller‐Institute for Plant Sciences Georg August University Göttingen Göttingen Germany; ^3^ Marbio UiT The Arctic University of Norway Tromsø Norway; ^4^ Centro Regional de Investigación y Desarrollo Sustentable de Atacama (CRIDESAT) Universidad de Atacama Copiapó Chile; ^5^ Institute of Geosciences University of Potsdam Potsdam Germany

**Keywords:** cell detachment, deep biosphere, DNA desorption, DNA extraction, intracellular DNA, low biomass

## Abstract

The simultaneous extraction of intracellular DNA (iDNA) and extracellular DNA (eDNA) can help to separate the living in situ community (represented by iDNA) from background DNA that originated both from past communities and from allochthonous sources. As iDNA and eDNA extraction protocols require separating cells from the sample matrix, their DNA yields are generally lower than direct methods that lyse the cells within the sample matrix. We, therefore, tested different buffers with and without adding a detergent mix (DM) in the extraction protocol to improve the recovery of iDNA from surface and subsurface samples that covered a variety of terrestrial environments. The combination of a highly concentrated sodium phosphate buffer plus DM significantly improved iDNA recovery for almost all tested samples. Additionally, the combination of sodium phosphate and EDTA improved iDNA recovery in most of the samples and even allowed the successful extraction of iDNA from extremely low‐biomass iron‐bearing rock samples taken from the deep biosphere. Based on our results, we recommend using a protocol with sodium phosphate in combination with either a DM (NaP 300 mM + DM) or EDTA (NaP + EDTA 300 mM). Furthermore, for studies that rely on the eDNA pool, we recommend using buffers solely based on sodium phosphate because the addition of EDTA or a DM resulted in a decrease in eDNA for most of the tested samples. These improvements can help reduce community bias in environmental studies and contribute to better characterizations of both modern and past ecosystems.

## Graphical abstract

The simultaneous extraction of intracellular DNA (iDNA) and extracellular DNA (eDNA) can be improved by using a highly concentrated sodium phosphate buffer with a detergent mix or EDTA. This improved protocol allows for higher recovery of iDNA from various environmental samples, including low‐biomass iron‐bearing rock samples, while buffers based solely on sodium phosphate are recommended for studies focusing on eDNA. These modifications may contribute to a more accurate characterization of modern and past ecosystems in environmental studies

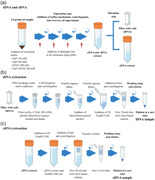

## 1 INTRODUCTION

Recent technological advancements in DNA sequencing have greatly contributed to our understanding of complex microbial communities in a variety of non‐extreme and extreme habitats, including for example, forest soil (Cardenas et al., 2015), permafrost (e.g., Yang et al., 2021), deep biosphere (e.g., Moreno‐Ulloa et al., 2019; Vuillemin et al., 2018), and desert environments (e.g., Genderjahn et al., 2021; Schulze‐Makuch et al., 2021). However, selecting the appropriate method for DNA extraction is an essential and sensitive step in molecular microbiology as DNA quality and quantity influence the success and reliability of every downstream analysis (Williamson et al., 2011), especially in the field of environmental microbiology (Tanase et al., 2015; Walden et al., 2017).

So far, most of the environmental studies based their interpretations and conclusions on the analysis of total DNA, which is the sum of all DNA pools present in one sample. However, total DNA represents, in general terms, two pools: the intracellular DNA (iDNA), which comes from intact living cells, and extracellular DNA (eDNA), which can persist in the environment and iis comprised of DNA that is not contained within an intact cellular membrane (i.e., dead communities or allochthonous sources; Alawi et al., 2014; Carini et al., 2016). This “dead” fraction can represent more than 40% of the total soil DNA pool (Ascher et al., 2009; Carini et al., 2016). Therefore, molecular biological analyses of DNA based on total DNA extractions can be biased toward higher diversity, causing significant misestimation of single taxa‐/taxon‐relative abundances leading to misinterpretation of specific environmental processes and their importance for the ecosystem (Carini et al., 2016; Lombard et al., 2011). Accordingly, the separation and extraction of iDNA could help to analyze the living fraction of the environmental sample, especially for low biomass environments where isolating RNA or other activity markers (e.g., intracellular ATP) is particularly challenging.

In this context, Alawi et al. (2014) developed a novel DNA extraction method that simultaneously separates iDNA and eDNA from the same sample. As the iDNA is extracted from intact cells, it provides information regarding the living and potentially active community, whereas the simultaneously separated eDNA bears the background of genetic information derived from dead organisms that can be used for studies on relic DNA (Ibáñez de Aldecoa et al., 2017). The iDNA and eDNA extraction protocol was initially developed for marine sediment samples but has also been successfully applied to different terrestrial environments, including soils from the Atacama Desert (Schulze‐Makuch et al., 2018; 2021) and different rock types (limestone, quartz‐rich shale, and quartz‐rich sandstone) from dryland in Namibia (Genderjahn et al., 2021).

It is important to consider that the iDNA and eDNA extraction procedure requires cells to be separated from the substrate before DNA extraction, leading to lower DNA yields in comparison to total DNA extraction methods which lyse the cells within the sample matrix (Williamson et al., 2011). Previous studies have only addressed DNA recovery problems associated with total DNA extraction from environmental samples and modifications were often required to achieve sufficient DNA yields, especially for low‐biomass samples (e.g., Barton et al., 2006; Lever et al., 2015). DNA sorption to soil particles has been successfully reduced in some studies using a highly concentrated phosphate buffer during DNA extraction (Direito et al., 2012; Lever et al., 2015). Additionally, adding EDTA can lead to the chelation of DNA‐adsorbing metal ions, thereby allowing for DNA extraction from volcanic ash soils (Rai et al., 2010). This approach has also been successfully used in fluorescence in situ hybridization applications by preventing the adsorption of DNA probes to sediment particles and thereby reducing background noise in the analysis (Morono et al., 2020).

On the other hand, to increase cell recovery, different extraction buffer modifications—including the use of different surfactants—have been applied (Kallmeyer et al., 2008; Williamson et al., 2011). For example, adding a detergent mix (DM) that includes EDTA, pyrophosphate, and Tween 80 in combination with methanol to a sediment sample has proven to increase cell recovery (Kallmeyer et al., 2008), which consequently can increase iDNA recovery. However, no further systematic studies on terrestrial samples have been conducted to improve the iDNA and eDNA extraction methods.

Our study combined existing approaches, including using a DM and EDTA to increase cell detachment and DNA recovery from the sample matrix during iDNA and eDNA extraction. The presented modifications were tested on different soil and rock samples that were taken along the Chilean Coastal Cordillera and aimed to improve iDNA recovery for a more precise characterization of the living community. This new method will help assess ecological questions in future environmental studies, especially when dealing with low‐biomass samples (data available in Medina & Horstmann et al., 2023).

## 2 METHODS

### 2.1 Sample material

**Figure A1:**
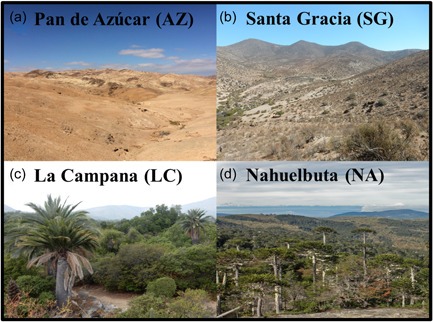
Picture of the location of the samples used in this study. (a) The northernmost site—Pan de Azúcar (AZ)—is characterized by arid conditions due to its location within the southern extent of the Atacama Desert. (b) Following the climate gradient to the south, Santa Gracia (SG) reflects a semi‐arid ecosystem, (c) La Campana (LC) is located in a Mediterranean climate, and (d) the southernmost site—Nahuelbuta (NA)—is characterized by temperate humid conditions.

During fieldwork in 2019 and 2020, soil and rock samples from different depths were taken from four sites along a climatic gradient in the Coastal Cordillera in Chile (Bernhard et al., 2018; Oeser et al., 2018) and were transported on an ice pack and stored at 4°C until processing. The northernmost site—Pan de Azúcar (AZ)—is characterized by arid conditions due to its location within the southern extent of the Atacama Desert. Following the climate gradient to the south, Santa Gracia (SG) reflects a semi‐arid ecosystem, La Campana (LC) is located in a Mediterranean climate, and the southernmost site—Nahuelbuta (NA)—is characterized by temperate humid conditions (pictures of all sites in Appendix Figure A1). For each site, a surface and a subsurface sample were processed (e.g., for Nahuelbuta: NA‐1 = Nahuelbuta surface sample, NA‐2 = Nahuelbuta subsurface sample). The soils were classified according to the Food and Agriculture Organization of the United Nations (FAO, 2006, see Bernhard et al., 2018) as follows: AZ—Regosol; SG—Cambisol; LC—Cambisol; NA—Umbrisol. Additionally, two samples from drill cores recovered in Santa Gracia (SG‐34 & SG‐59) were processed to assess the applicability of the iDNA and eDNA protocol to granitic rock samples from deep biosphere environments. An overview of the processed samples is shown in Table 1.

**Table 1:** Overview of samples used in this study.

Sample name	Latitude	Longitude	Soil classification	Depth	Characteristic/sample type
Pan de Azúcar 1 (AZ‐1)	–26.301967,	–70.458433	Regosol	0–5 cm	Surface; soil sample
Pan de Azúcar 2 (AZ‐2)	–26.304400	–70.455867	Regosol	40–60 cm	Subsurface; soil sample
Santa Gracia 1 (SG‐1)	–29.759037	–71.160226	Cambisol	0–5 cm	Surface; soil sample
Santa Gracia 2 (SG‐2)	–29.759037	–71.160226	Cambisol	40–60 cm	Subsurface; soil sample
Santa Gracia 34 (SG‐34)	–29.759414	–71.160322	‐	39.70–1.20 m	Subsurface; rock core
Santa Gracia 59 (SG‐59)	–29.759414	–71.160322	‐	74.20–75.70 m	Subsurface; rock core
La Campana 1 (LC‐1)	–33.02833	–71.04370	Cambisol	0–5 cm	Surface; soil sample
La Campana 2 (LC‐2)	–33.02833	–71.04370	Cambisol	40–60 cm	Subsurface; soil sample
Nahuelbuta 1 (NA‐1)	–37.79381	–72.95043	Umbrisol	0–5 cm	Surface; soil sample
Nahuelbuta 2 (NA‐ 2)	–37.79381	–72.95043	Umbrisol	5–10 cm	Subsurface; soil sample

### 2.2 Intra‐ and eDNA separation and extraction

**Figure 1:**
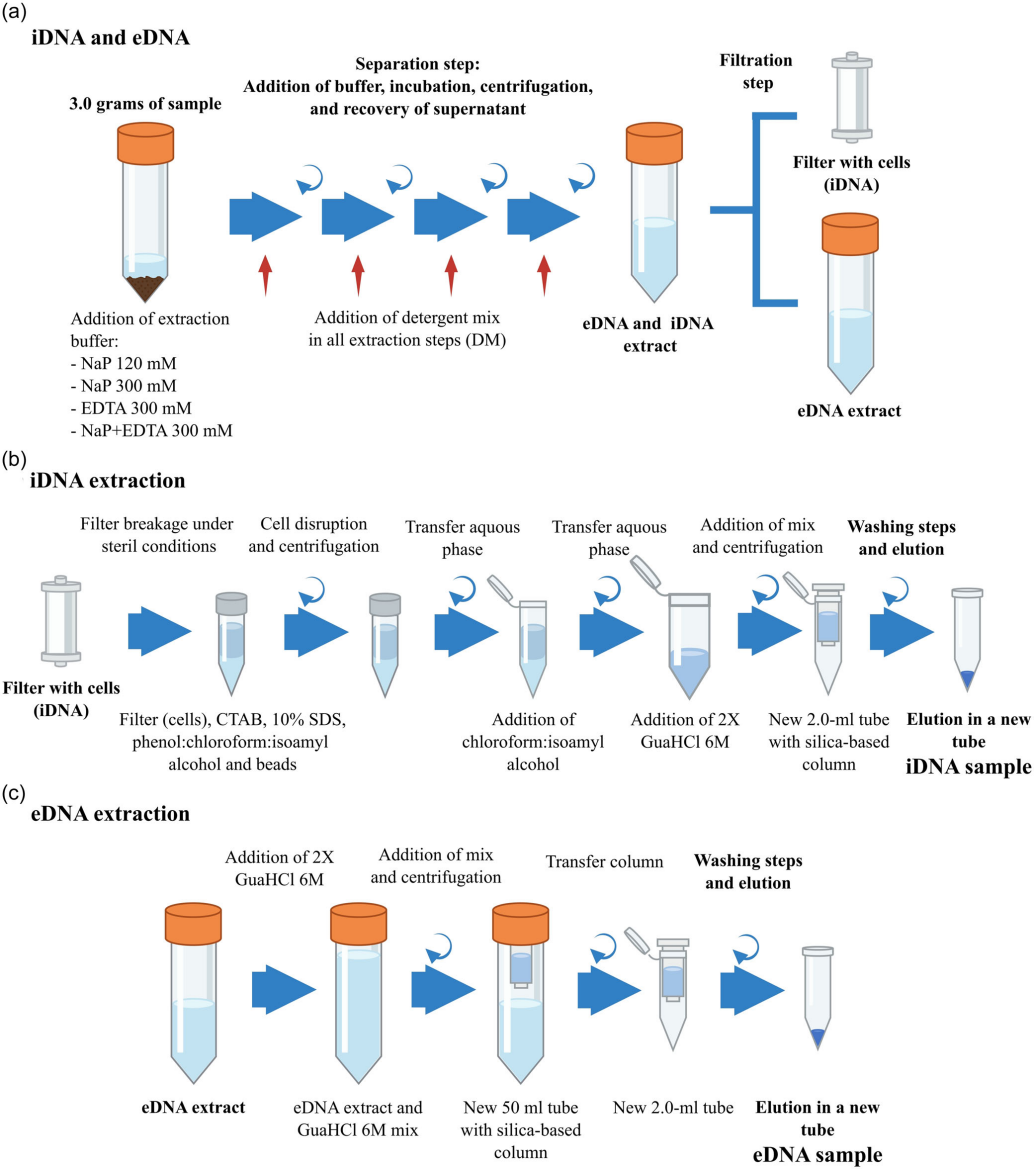
Scheme of eDNA and iDNA separation/extraction method. (a) General workflow of eDNA and iDNA separation; (b, c) extraction of iDNA (b) and eDNA (c) following the methods described in this work. Sodium phosphate buffer of 120 mM (NaP 120 mM), sodium phosphate buffer of 300 mM (NaP 300 mM), EDTA buffer of 300 mM (EDTA 300 mM), and sodium phosphate–EDTA buffer of 300 mM (NaP+EDTA 300 mM). Detergent mix (DM).

The DNA from all samples was extracted in triplicates using a protocol based on the iDNA and eDNA extraction protocol developed by Alawi et al. (2014). All modifications and alternative setups compared with the latter method are described below (Figure 1). Three grams of sample material were mixed with 6 mL of 120‐mM sodium phosphate (NaP) buffer pH 8.0 (400 mL of 120‐mM Na_2_HPO_4_ + 29.2 mL of 120‐mM NaH_2_PO_4_, sterile filtered over a 0.2‐µm PES filter and autoclaved) or with alternative buffers (see below) in sterile 50‐mL tubes. Samples were placed on ice for 1 min, followed by agitation on a horizontal shaker for 5 min at 150 rpm. After cooling the samples on ice for 1 min, the shaking step was repeated. The resulting slurry was centrifuged for 10 min at 500*g*. The supernatant containing the eDNA and iDNA fractions was collected in a new sterile 50‐mL tube and stored on ice. Another 3 mL of buffer was added to the remaining slurry, followed by an additional round of agitation on the shaker, subsequent centrifugation, and supernatant collection. The entire procedure was repeated twice (total: 4 times). About 15 mL of iDNA‐ and eDNA‐containing supernatant was collected. To separate the iDNA (enclosed in the intact cells) from the eDNA, the extract was filtered through a 0.22‐µm Sterivex™ filter unit (Millipore) using a sterile syringe, thereby leaving the cells attached to the filter membrane while the flow‐through that contained the eDNA was collected for further extraction. Finally, the filter was rinsed with 3 mL buffer to wash off the remaining eDNA.

To extract the iDNA, the filters containing the cells were cracked open under sterile conditions, and the filter membrane was cut off using a sterile scalpel and tweezers and then transferred to a 2.0‐mL screw‐cap tube along with glass‐ and zirconia beads. The iDNA was extracted from the filters using CTAB buffer, phenol:chloroform:isoamyl alcohol (25:24:1), and 10% SDS (modified from Nercessian et al., 2005). After centrifugation at 16,000*g* and 4°C for 10 min, the aqueous phase was transferred to a new tube and mixed with the same volume of chloroform:isoamyl alcohol (24:1), and the centrifugation step was repeated to remove any phenol residues. The aqueous phase was again transferred to a new 2.0‐mL tube and mixed with 1.2 mL (2 volumes) of guanidine hydrochloride (GuaHCl, 6 M in x1 TE buffer pH 6.7). The GuaHCl‐mix was transferred onto a silica‐based spin column in a sterile 2 mL tube (Zymo Research) and centrifuged at 5000*g* for 1 min at room temperature. The flow‐through was discarded, and the step was repeated. Then, the silica filter was washed twice by adding 500 µL of washing buffer (50% EtOH, 125 mM NaCl, 10 mM tris, 1 mM EDTA, pH 8.0) and centrifuged at 5000*g*. To remove any remaining washing buffer, the column was dried via centrifugation at 5000*g* for 2 min and transferred to a new sterile 2.0‐mL tube. Finally, the iDNA was eluted in 100 µL of 10 mM Tris buffer (pH 8.0) via centrifugation at 5000*g* for 2 min.

For the eDNA extraction, the collected flow‐through that contained the eDNA was thoroughly mixed with 30 mL (2 volumes) of GuaHCl, transferred to a sterile 50‐mL tube that contained a silica‐based spin column with an adaptor for 50‐mL tubes (Zymo Research), and centrifuged at 500*g* for 5 min at room temperature. The flow‐through was discarded, and the step was repeated. Afterward, the column was washed twice by adding 500 µL of washing buffer, centrifuged at 500*g*, and transferred to a sterile 2.0‐mL tube. Then, the column was dried via centrifugation at 5000*g* for 2 min and transferred to a sterile 2.0‐mL tube. Finally, the eDNA was eluted as described above.

A negative control was included in all DNA extractions with all methods: one negative control for eDNA extraction, two negative controls for iDNA extraction, one negative control for the respective filter of the negative control during the separation step, and one extra negative control to check all the reagents during iDNA extraction.

### 2.3 Alternative buffers

To systematically analyze and improve the method described by Alawi et al. (2014), we used three different alternative buffers, including negative controls for all of the setups. In addition to the 120‐mM NaP buffer from the original protocol, we used a 300‐mM NaP buffer (400 mL of 300‐mM Na_2_HPO_4_ + 29.2 mL of 300‐mM NaH_2_PO_4_), 300 mM of EDTA, and a mix of both NaP and EDTA (300 mM). All buffers were adjusted to pH 8.0, passed through a 0.2‐µm filter, and autoclaved.

Additionally, we tested all buffers in combination with a DM—as described in Kallmeyer et al. (2008)—that contained 100 mM of EDTA, 100 mM of sodium pyrophosphate, 1% (v/v) Tween 80, and 1% (v/v) methanol. The mix was added in all separation steps (four times) of the iDNA and eDNA extraction protocol, 600 μL of DM and 600 μL of methanol were added in the first separation step and 300 μL in each of the remaining three steps.

### 2.4 Quantification of DNA pools

The concentration of both DNA pools was quantified fluorometrically using the Qubit™ dsDNA HS Assay Kit (Thermo Fisher Scientific). Additionally, quantitative PCR (qPCR) was performed using a CFX Connect Real‐Time PCR detection system (BioRad). The qPCR was performed in 20 µL of master mix that contained 10 µL of KAPPA Hifi SYBR Mix 1x (Qiagen), 0.4 µL each of 10‐µM universal primers 341 F (5′‐CCTACGGGAGGCAGCAG‐3′) and 534 R (5′‐ATTACCGCGGCTGCTGG‐3′) (Muyzer et al., 1993), 5.2 µL of PCR‐grade H_2_O, and 4 µL of DNA template. The following cycling parameters were used: initial denaturation at 95°C for 3 min followed by 40 cycles (95°C, 3 s of denaturation; 60°C, 20 s of annealing; 72°C, 30 s of elongation; 80°C, 3 s of plate reading). All samples were analyzed by running three technical replicates. A standard with a known concentration (2.5 × 10^8^ gene copies) of a 16 S rRNA gene PCR fragment of *Bacillus subtilis* was used to generate a standard curve via serial dilution (10^1^–10^7^ gene copies) and to calculate the efficiency (>90% to <110%) using the BioRad CFX software. Corresponding extraction negative controls and qPCR nontemplate controls were included in the analysis. A melting curve analysis was conducted at the end of each run to identify any nonspecific DNA amplification.

### 2.5 Cell counting

For the cell counting on the test sample (AZ‐2), 3 grams of sample material were extracted with the separation step in the iDNA and eDNA extraction protocol (see above 3.2; Figure 1a), omitting the filtration step. The cells from the “iDNA and eDNA extract” were fixed with 10% Paraformaldehyde in a 1:1 ratio and incubated overnight at 4°C. Afterward, 10 mL of the fixed solution was filtered using a 0.2 μm filter (Anodisc™ 25, Whatman™), and the filters were stored at −20°C.

The cells were stained with SYBR Green I according to the protocol of Noble and Fuhrman (1998) with 0.1% p‐phenylenediamine as an antifading agent. Cells were counted using epifluorescence microscopy, using a blue filter set (Leica DM1000 Fluorescence Microscope Filter System I3) and covering 200 fields of view (FOV) or until counting 200 cells (Kallmeyer et al., 2008). The cell number was calculated with the following formula:

$$cell\;\mathrm{count}\;\left(\mathrm{cell}\;g^{-1}\right)=\frac{20106\times total\;\mathrm{cells}\;\mathrm{counted}\times \mathrm{dilution}\;\mathrm{factor}}{FOV\times \;\mathrm{sample}\;\mathrm{weight}}$$

Where the factor “20106” reflects the area of the filter and FOV indicates the number of counted fields.

### 2.6 Additional tests—samples spiked with genomic DNA

Different tests were performed to monitor the transfer of eDNA into the iDNA pool. Firstly, to evaluate the attachment of eDNA to the filter membrane, 1 µg of genomic DNA was added to 15 mL of 120 mM NaP buffer and incubated for 1 h on ice. Afterward, the sample was filtered, and the iDNA and eDNA were extracted, as mentioned before (Section 3.2). The extractions were done in triplicates.

The transference of eDNA through fine mineral particles to the iDNA pool was checked by spiking 1 µg of genomic DNA into 3 g of DNA‐free combusted sand (4.5 h at 450°C, washed twice with NaP buffer, centrifuged for 10 min at 500*g* and the supernatant was removed as described by Alawi et al., 2014) and the procedure described in Section 3.2 was performed. To compare the recovery rate of iDNA and eDNA, the DNA‐free combusted sand was also extracted without adding DNA. The extractions were done in triplicates.

Additionally, 500 ng of genomic DNA was spiked into 3 g of the test sample (AZ‐2), and then the soil material was extracted using the selected setups: NaP 120 mM, NaP 300 mM + DM, and NaP + EDTA 300 mM. The recovery was compared with the samples extracted without added DNA. All tests were performed in triplicates.

### 2.7 Data preparation and statistical analysis

The gene copy number was calculated based on the standard curve and divided by the amount of soil/rock samples in grams (gene copy numbers per gram sample). The mean of the three replicates and the standard deviation were calculated for visualization. An improvement factor was calculated for all samples by dividing the yields of the alternative methods by the yields of the original method, with values above 1 indicating an improvement compared with the method developed by Alawi et al. (2014). To visualize improvements in samples from which no DNA could be extracted using the buffer NaP 120 mM (e.g., SG‐34, SG‐59), gene copy numbers of the corresponding negative control and the detection limit of the Qubit™ dsDNA HS Assay Kit (0.01 ng µL^−1^) were used. Additionally, a spiked/nonspiked ratio was calculated for the spiked experiment by dividing the values (raw qPCR data or gene copy numbers) of the spiked samples versus the non‐spiked samples. Values above 1 indicate an increase in DNA recovery due to adding DNA. All plots were generated with R statistical software (R Core Team, 2022) using the ggplot2 package (Wickham, 2016). Statistical analyses were performed using a Friedman test to check significant differences between the modifications and the original method. *p* Values below 0.05 were considered statistically significant.

## 3 RESULTS

### 3.1 DNA recovery with alternative buffers and the addition of DM on the test sample AZ‐2

To optimize iDNA recovery during iDNA and eDNA extraction, different modifications in the separation step of the extraction protocol were tested on the Pan de Azúcar subsurface sample (AZ‐2). This low biomass sample (3.0 × 10^3^ cell g^−1^, Appendix Table A5) was taken from the subsurface of the driest site of the climatic gradient in the Coastal Cordillera in Chile, representing extremely challenging DNA extraction conditions. Besides the sodium buffer (120 mM) from Alawi et al. (2014), three alternative buffers (without additional DM) were tested: a sodium phosphate buffer of 300 mM (NaP 300 mM), an EDTA buffer of 300 mM (EDTA 300 mM), and a sodium phosphate–EDTA buffer of 300 mM (NaP+EDTA 300 mM). Each of the four buffers was further tested in combination with a DM (100 mM of EDTA, 100 mM sodium pyrophosphate, 1% (v/v) Tween 80, and 1% methanol (v/v)).

**Figure 2:**
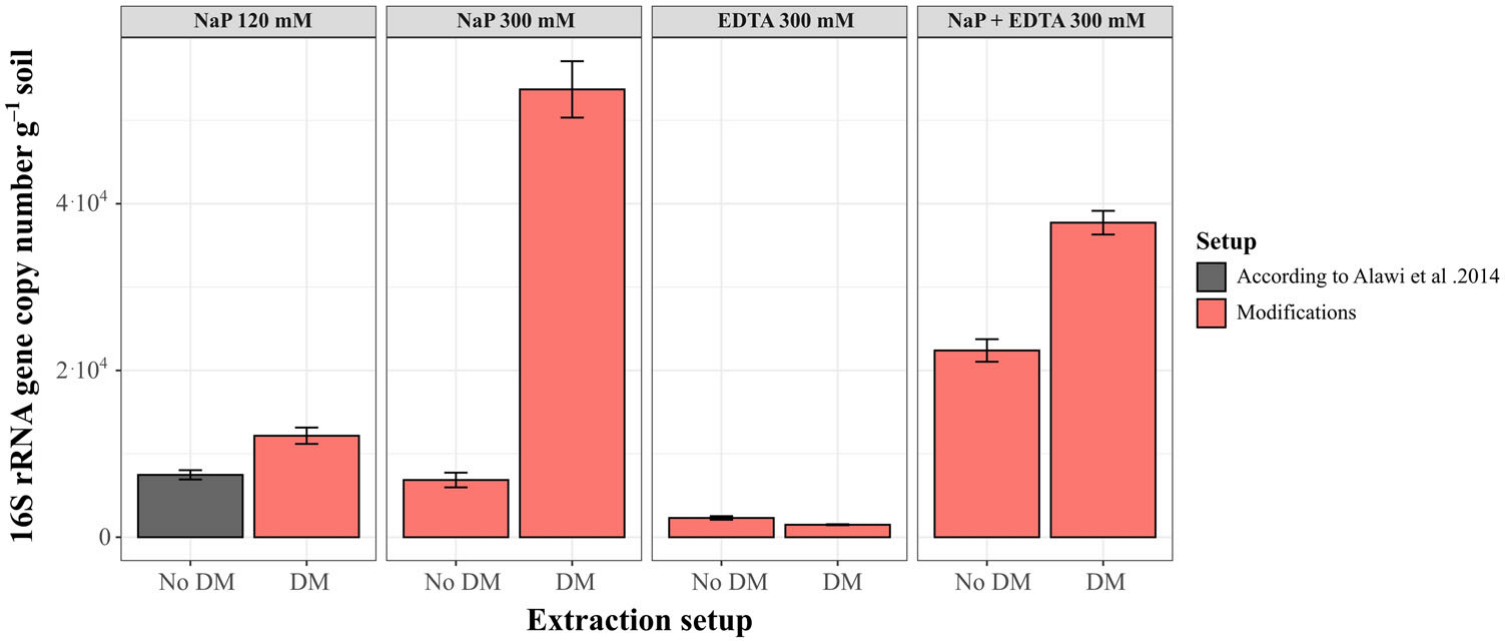
16S rRNA gene copy numbers of iDNA from the Pan de Azúcar soil subsurface sample (AZ‐2) using four different buffers: a sodium phosphate buffer of 120 mM (NaP 120 mM, according to Alawi et al. (2014) in gray), a sodium phosphate buffer of 300 mM (NaP 300 mM), an EDTA buffer of 300 mM (EDTA 300 mM), and a sodium phosphate–EDTA buffer of 300 mM (NaP + EDTA 300 mM). No DM—no detergent mix added, DM ‐ detergent mix added.

The measured DNA concentrations for the subsurface sample of AZ‐2 were mainly below the detection limit of 0.01 ng µL^–1^ (Appendix Tables A3 and A4), regardless of the buffer setups used. Only the eDNA extracted with the NaP 120 mM and 300 mM buffers recovered measurable amounts of DNA (0.016 ng µL^−1^ with NaP 120 mM and 0.020 ng µL^−1^; Appendix Tables A3 and A4). Therefore, only 16 S rRNA gene copy numbers were considered when evaluating the different extraction setups (Figure 2).

Extracting with the NaP 120 mM buffer, Alawi et al. (2014) led to iDNA yields of around 7.5 × 10^3^ gene copies g^−1^ soil (Appendix Table A4). Generally, the change of extraction buffer and the addition of DM improved iDNA yield for most of the alternative setups. The most significant improvement compared with the NaP 120 mM buffer was achieved using the NaP 300 mM buffer with the DM, which reached 5.4 × 10^4^ gene copies g^−1^ soil—seven times higher than with the NaP 120 mM method (Figure 2). The NaP + EDTA 300 mM buffer improved iDNA yields by 3 times without the DM (2.2 × 10^5^ gene copies g^−1^ soil) and up to 5 times with the DM (3.8 × 10^5^ gene copies g^−1^ soil; Figure 2). A slight improvement was achieved when adding the DM to the NaP 120 mM buffer increasing gene copy numbers from 7.5 × 10^3^ to 1.2 × 10^4^. The use of the NaP 300 mM buffer without DM (no DM) achieved values of 6.9 × 10^3^ gene copy numbers, slightly below the yields of the NaP 120 mM buffer. The setups with only EDTA as extraction buffer resulted in a loss of iDNA, decreasing to around 2.3 × 10^3^ gene copies g^−1^ soil without DM and even further decreasing to 1.5 × 10^3^ gene copies g^−1^ soil after adding the DM (Figure 2, Appendix Table A1).

**Figure 3:**
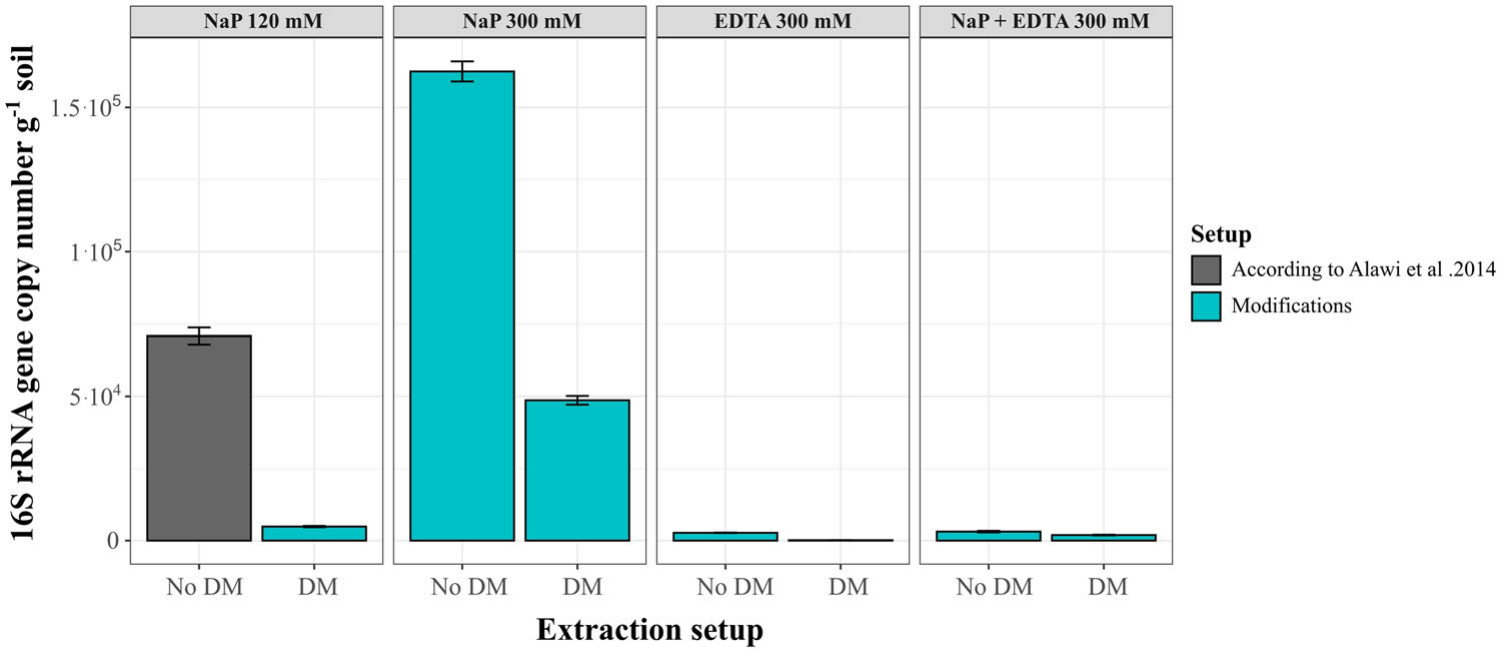
16S rRNA gene copy numbers of eDNA from the Pan de Azúcar soil subsurface sample (AZ‐2) using four different buffers: a sodium phosphate buffer of 120 mM (NaP 120 mM, according to Alawi et al. (2014) in gray), a sodium phosphate buffer of 300 mM (NaP 300 mM), an EDTA buffer of 300 mM (EDTA 300 mM), and a sodium phosphate–EDTA buffer of 300 mM (NaP+EDTA 300 mM). No DM, no detergent mix added; DM, detergent mix added.

The eDNA yield from AZ‐2 only improved after using the higher concentrated NaP 300 mM buffer reaching values of 1.6 × 10^5^ gene copies g^−1^ soil which is twice as high as the NaP 120 mM with values around 7.1 × 10^4^ gene copies g^−1^ soil. While the NaP 300 mM buffer with DM gave similar eDNA yields to the NaP 120 mM setup of 4.9 × 10^4^ gene copies g^−1^ soil the eDNA yield decreased for all other setups. Extraction setups containing EDTA (EDTA 300 mM and NaP+EDTA 300 mM) decreased eDNA recovery by at least one order of magnitude (Figure 3, Appendix Table A2).

Additionally, cell counting was also performed on the AZ‐2 sample to verify and compare the iDNA yield with cell numbers (see Appendix Table A5). The results showed x4 higher cell numbers when using the NaP 300 mM buffer plus DM (1.1 × 10^4^ cells g^−1^) in comparison to the NaP 120 mM buffer (3.01 × 10^3^ cell g^−1^), which is supported by a similar increase in the qPCR data.

### 3.2 iDNA ‐ additional tests to exclude eDNA cross‐contamination

Another important factor to consider is whether there is eDNA cross‐contamination, meaning that the iDNA yield can be increased by the transfer of eDNA to the iDNA pool,  attaching directly to the filter material or by fine mineral particles that are retained by the filter membrane during the filtration step. Firstly, we analyzed the attachment of eDNA to the filter membrane by adding purified genomic DNA. The results showed that eDNA did not attach to the filter since the signal measured in the iDNA pool was similar to the negative control (Table 2, Appendix Table A6). We tested DNA‐free combusted sand 1 μg with spiked genomic DNA to check for eDNA being transferred to the iDNA pool by the attachment to fine mineral particles. The results indicated that no or only small DNA amounts were transferred since the values (spiked/nonspiked) were close to the respective control (Table 2). To further validate this, we spiked the test sample AZ‐2 with 500 ng genomic DNA. The results showed that only the eDNA ratio (spiked/non‐spiked) increased up to 7−8 times (except for NaP 300 mM + DM) which indicated that the spiked DNA goes into the eDNA as expected (Table 3, Appendix Table A7). On the other hand, the spiked/nonspiked ratio for the iDNA pool was around 1, confirming that eDNA is not transferred into the iDNA pool by attaching to the filter material or mineral particles. Only the sample extracted with the NaP 300 mM + DM setup did not show the expected ratios. For the eDNA and iDNA, the ratio was 0.55 and 0.22, respectively, since we found that the spiked surprisingly sample showed a lower DNA recovery in comparison with the non‐spiked sample (Table 3).

**Table 2:** Spiked experiment with the Buffer NaP 120 mM and sand. eDNA and iDNA yields, with raw qPCR data, of nonspiked and 1 μg genomic DNA spiked with the Buffer NaP 120 mM and free‐DNA combusted sand.

Sample	eDNA/iDNA nonspiked	eDNA/iDNA; +1µg genomic DNA—Spiked	Ratio spiked/nonspiked
Buffer NaP 120 mM—eDNA	16.09	3010.62	189.17
Sand—eDNA	14.99	7211.11	481.93
Buffer NaP 120 mM—iDNA	27.87	41.17	1.49
Sand—iDNA	31.59	66.82	2.13

**Table 3:** Spiked experiment with AZ‐2.

**DNA pool**	**Setup**	**eDNA/iDNA nonspiked**	**eDNA/iDNA** + **500 ng genomic DNA—spiked**	**Ratio spiked/nonspiked**
AZ‐2 eDNA	NaP 120 mM	70,828.40	595,045.60	8.40
	NaP 300 mM + DM	48,646.45	26,713.86	0.55
	NaP + EDTA 300 mM	3120.95	22,250.68	7.13
AZ‐2 iDNA	NaP 120 mM	7482.90	6359.04	0.85
	NaP 300 mM	53,715.81	10,461.89	0.19
	NaP + EDTA 300 mM	22,399.97	23,303.19	1.04

*Note*: eDNA and iDNA yields, yields, in 16S gene copy number g‐1, of the nonspiked and 500 ng genomic DNA spiked with the test sample AZ2 using “NaP 120 mM”. “NaP 300 mM + DM” and “NaP+EDTA 300 mM” setups.

### 3.3 Evaluation of the improved extraction methods on soil and drill core samples

#### 3.3.1 NaP 300 mM + DM extraction setup

**Figure 4:**
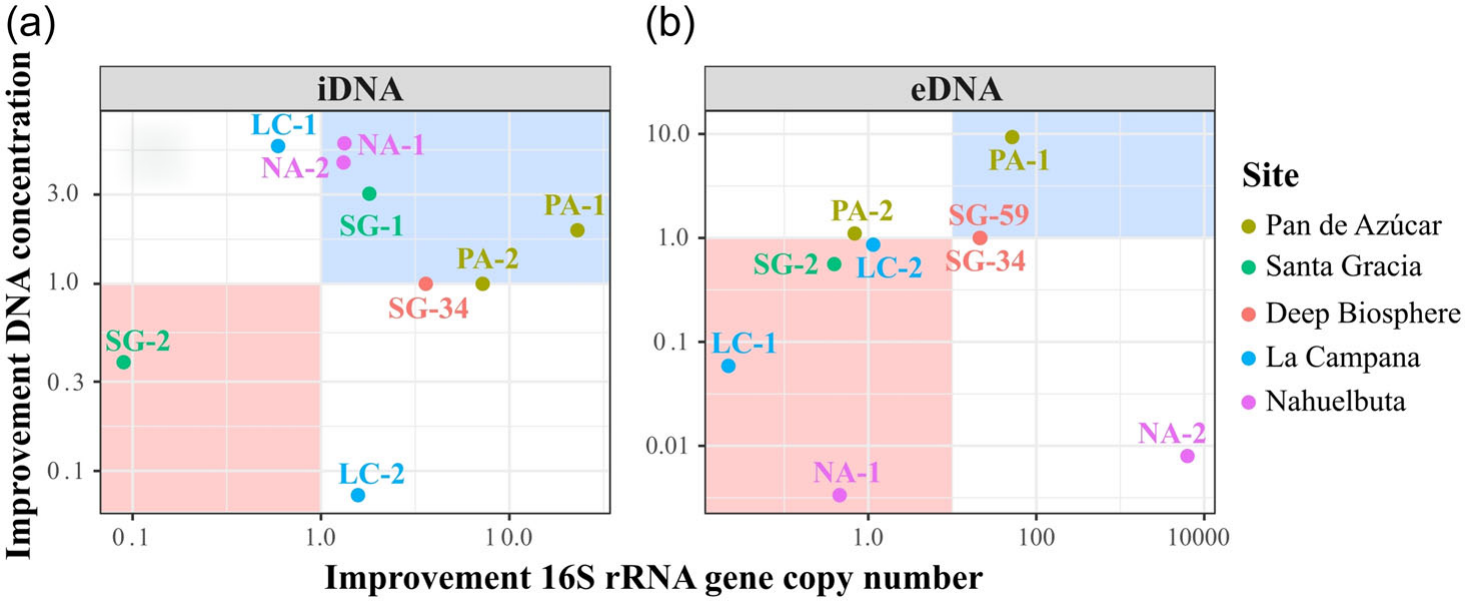
Improvement factors with the 300 mM sodium phosphate buffer with detergent mix (NaP 300 mM + DM) setup. Improvement factors of concentrations and 16S rRNA gene copy numbers for iDNA (a) and eDNA (b) for the different samples: Pan de Azúcar (AZ‐1, AZ‐2), Santa Gracia (SG‐1. SG‐2), the deep biosphere (SG‐34, SG‐59), La Campana (LC‐1, LC‐2) and Nahuelbuta (NA‐1, NA‐2). Values > 1 on the x‐ and y‐axes indicate an improvement in gene copy numbers or DNA concentrations compared with the original NaP 120‐mM method by Alawi et al. (2014). Samples within the red area showed a decrease in DNA recovery, while those within the blue area showed an increase in both DNA concentrations and gene copy numbers. Scales are logarithmic.

To verify the NaP 300 mM buffer + DM setup, we tested it on a variety of environmental samples, including Nahuelbuta (NA‐1 and NA‐2), La Campana (LC‐2), Santa Gracia (SG‐1, SG‐2, SG‐34, and SG‐59), and Pan de Azúcar (AZ‐1). DNA concentration and gene copy number were used to evaluate the improvement of iDNA and eDNA extraction efficiency (Figure 4). The improvement was calculated for each sample by dividing the gene copy numbers or DNA concentrations recovered with the NaP 300 mM + DM by the values obtained with the NaP 120 mM buffer (Figure 4). For the iDNA, SG‐59 as well as for the eDNA the surface samples SG‐1 could not recover measurable or amplifiable DNA using the alternative or the NaP 120 mM setups, thus, these samples were removed from the plots (Figures 4a,b).

Except for the Santa Gracia subsurface sample (SG‐2), all samples showed an increase in iDNA recovery reflected by an improvement of either DNA concentration or gene copy number using the NaP 300 mM + DM. This setup was statistically superior (*p* < 0.05) to the NaP 120 according to the Friedman test (Appendix Figure A3).

**Figure A2:**
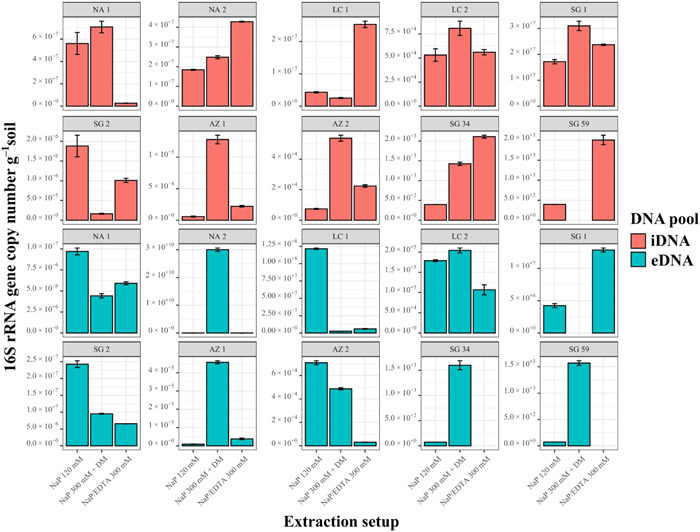
16S rRNA gene copy numbers using four different buffers: a sodium phosphate buffer of 120 mM (NaP 120 mM, according to Alawi et al., 2014), a sodium phosphate buffer of 300 mM (NaP 300 mM), and a sodium phosphate–EDTA buffer of 300 mM (NaP + EDTA 300 mM). 16 S rRNA gene copy numbers for iDNA and eDNA for the different samples: Pan de Azúcar (AZ‐1 and AZ‐2), Santa Gracia (SG‐1 and SG‐2), the deep biosphere (SG‐34 and SG‐59), La Campana (LC‐1 and LC‐2) and Nahuelbuta (NA−1 and NA‐2). DM, detergent mix added.

The alternative setup increased the iDNA recovery of both Pan de Azúcar samples (AZ‐1, AZ‐2; Figure 4a). The surface sample (AZ‐1) showed a 23‐fold increase in DNA concentration than the subsurface sample (AZ‐2) with 5.8 × 10^3^ to 1.3 × 10^5^ gene copies g^–1^ soil (Figure 4a, Appendix Table A3 and Figure A2). The surface sample in Santa Gracia (SG‐1) increased threefold (i.e., from 1.35 ng µL^–1^ to 4.10 ng µL µL^–1^; Appendix Table A3), which corresponded to about a doubling of 16 S rRNA gene copy numbers (Figure 4a) from 1.7 × 10^7^ to 3.1 × 10^7^ gene copies g^–1^ soil (Appendix Table A3 and Figure A2). Additionally, the deep biosphere sample SG‐34 yielded amplifiable DNA of 1.4 × 10^3^ gene copies g^–1^ rock after extraction with NaP 300 mM + DM (Appendix Table A3 and Figure A2). Small improvements with this buffer were observed for both Nahuelbuta samples (NA‐1 and NA‐2) and the La Campana subsurface sample (LC‐2; Figure 4a).

Compared with the iDNA, eDNA recovery from the samples was generally less improved by the alternative buffers (Figure 4b) as is also shown using the Friedman test where no significant differences were found with the different setups (Appendix Figure A4). However, the NaP 300 mM buffer plus DM (NaP 300 mM + DM) achieved an improvement for the Nahuelbuta subsurface sample (NA‐2), the Pan de Azúcar surface sample (AZ‐1), and the two deep biosphere samples from Santa Gracia (SG‐34 and SG‐59; Figure 4b).

For the Nahuelbuta sample, eDNA increased by a factor of 6,250 (Figure 4b), or from 4.8 × 10^6^ to 3.0 × 10^10^ gene copies g^–1^ soil (Appendix Table A4 and Figure A2). However, this increase is not consistent with the measured DNA concentrations, which dropped from 53 to 0.43 ng µL^–1^ (Appendix Table A4). For the Pan de Azúcar surface sample (AZ‐1), the use of the NaP 300 mM + DM setup resulted in a 50‐fold higher eDNA yield (Figure 4b). Moreover, for samples SG‐34 and SG‐59, which showed no measurable eDNA when using the NaP 120 mM buffer, gene copy numbers of more than 1.5 × 10^3^ g^–1^ rock were achieved using the NaP 300 mM + DM extraction setup (Appendix Table A4 and Figure A2). This corresponds to an improvement of at least 20‐fold (Figure 3b), considering the detection limit (i.e., 396 gene copies g^–1^ soil) of the qPCR run for the SG‐34 and SG‐59 samples, which had been extracted with the NaP 120 mM buffer.

#### 3.3.2 NaP+EDTA 300 mM extraction setup

**Figure 5:**
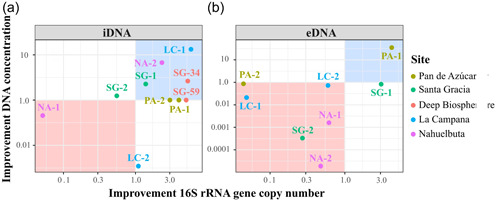
Improvement factors with the 300 mM sodium phosphate buffer plus 300 mM EDTA (NaP + EDTA 300 mM) setup. Improvement factors of concentrations and 16S rRNA gene copy numbers for iDNA (a) and eDNA (b) for the different samples: Pan de Azúcar (AZ‐1, AZ‐2), Santa Gracia (SG‐1. SG‐2), the deep biosphere (SG‐34, SG‐59), La Campana (LC‐1, LC‐2) and Nahuelbuta (NA‐1, NA‐2). Values > 1 on the x‐ and y‐axes indicate an improvement either for gene copy numbers or for DNA concentrations compared with the original NaP 120‐mM method by Alawi et al. (2014). Samples within the red area showed a decrease in DNA recovery, while those within the blue area showed an increase in both DNA concentrations and gene copy numbers. Scales are logarithmic.

Additionally, we tested an EDTA‐based buffer, the NaP+EDTA 300 mM extraction setup without DM, on all samples (Figure 5). Using this setup, the iDNA of the test sample AZ‐2 increased from 7.5 × 10^3^ to 2.2 × 10^4^ gene copies g^–1^ soil (3 times higher, Figure 5a, Appendix Table A3 and Figure A2) which was also shown by the increase in cell numbers from 2.97 × 10^3^ to 2.82 × 10^4^ (around nine times higher, Appendix Table A5).

Using the NaP + EDTA 300 mM extraction setup, the Pan de Azúcar surface sample (AZ‐1) showed a 4‐fold iDNA improvement compared with the NaP 120‐mM method and increased from 5.8 × 10^3^ to 2.1 × 10^4^ gene copies g^–1^ soil (Figure 5a). Moreover, the Nahuelbuta subsurface (NA‐2) sample doubled iDNA yields (Figure 5a) from 1.8 × 10^7^ to 4.7 × 10^7^ gene copies g^–1^ soil. This difference became even more apparent in the measured iDNA concentration, which rose more than 5 times from 1.16 to 6.10 ng µL^–1^ (Figure 5b, Appendix Table A3, and Figure A2). An important improvement with this extraction setup was achieved for the two deep biosphere samples from Santa Gracia (SG‐34 and SG‐59), for which no DNA could be extracted using NaP 120 mM. These samples yielded enough iDNA to be amplified and quantified by qPCR with values of around 2 × 10^3^ gene copies g^–1^ rock (Appendix Table A3 and Figure A2). This shows that the NaP + EDTA 300‐mM buffer led to the best extraction results for the deep biosphere samples analyzed in this study.

For the NaP+EDTA 300‐mM setup, only the eDNA from the Pan de Azúcar surface sample (AZ‐1) and the Santa Gracia surface sample (SG‐1) showed improvements. For AZ‐1 eDNA, recovery was 4‐fold higher (Figure 5b), increasing from 9.1 × 10^3^ to 3.8 × 10^4^ gene copies g^–1^ soil (Appendix Table A4 and Figure A2), whereas for SG‐1, a 3–fold improvement was observed (Figure 5b), with gene copy numbers of up to 1.3 × 10^7^ g^–1^ (Appendix Table A4 and Figure A2). For the NA‐1 and LC‐1 surface samples and the SG‐2 and AZ‐2 subsurface samples, eDNA yields decreased when using the alternative extraction setups, as shown by the values < 1 in Figure 5b.

## 4 DISCUSSION

### 4.1 Modifications on the iDNA and eDNA method

Many studies have tried to improve DNA extraction in the face of low‐biomass conditions or DNA adsorption (Barton et al., 2006; Direito et al., 2012; Lever et al., 2015; Williamson et al., 2011). Further, the separate extraction of the iDNA and eDNA pool from the same sample is a unique approach that has thus far only been applied in a few studies in extreme terrestrial ecosystems (e.g., Genderjahn et al., 2021; Schulze‐Makuch et al., 2018, 2021). In this approach, the most critical processes that influence its success are the detachment of cells and the desorption of DNA from the sample matrix.

In this context, cell detachment from the sample matrix requires that fundamental parameters, such as physical and chemical dispersion, are optimized (Williamson et al., 2011). Physical dispersion refers to the physical/mechanical process of releasing cells from the sample matrix. Our work avoided mechanical blending and heavy physical shaking by vortexing or ultrasonic treatment to minimize the risk of cell lysis. Instead, we chose a gentle incubation on an orbital shaker, using four washing steps that could recover over 90% of the eDNA as described by Alawi et al. (2014).

In terms of chemical dispersion, however, no clear consensus has yet been reached, and various specific extraction buffers have been described (Lever et al., 2015; Williamson et al., 2011). Support has been found for using phosphate buffers (Alawi et al., 2014; Corinaldesi et al., 2005; Lever et al., 2015; Ogram et al., 1987), and particularly for highly concentrated phosphate buffers (Direito et al., 2012; Lever et al., 2015). In addition, using EDTA has successfully prevented DNA adsorption (Rai et al., 2010). Moreover, mild detergents have been reported to be pessential to the complete dispersion of soil aggregates and to the detachment of cells from soil surfaces (Williamson et al., 2011). We, therefore, studied the effects of different concentrated phosphate buffers and EDTA, both with and without adding a DM.

### 4.2 Cell detachment: iDNA yields

Various mechanisms keep cells attached to surfaces, such as direct adhesion (Corinaldesi et al., 2005) and biofilm formation, which are involved in the colonization of mineral surfaces (Costa et al., 2018; Fletcher & Floodgate, 1973). Therefore, a major challenge in successfully recovering iDNA involves the detachment of cells from the sample matrix while maintaining cell integrity. Adding a DM in the washing steps successively increased iDNA recovery. This finding is consistent with observations in which the DM contributed to increased cell detachment for quantitative analysis, such as cell counting (Kallmeyer et al., 2008). Notably, our results indicate that the use of a DM has a variable effect on iDNA recovery depending on whether it is used in combination with a phosphate buffer (NaP 120 mM, NaP 300 mM), an EDTA buffer (EDTA 300 mM), or a mixture of both (NaP+EDTA 300 mM). For most of our results, we observed an increase in iDNA recovery when the DM was added. The most remarkable improvements occurred when using the detergent mix in combination with sodium phosphate (NaP 120 mM, NaP 300 mM) or when using sodium phosphate mixed with EDTA 300 mM (NaP + EDTA 300 mM). This indicates that phosphate constitutes an important part of the functionality of the DM.

Detergents are commonly used in total DNA extractions to lyse cells for a higher DNA recovery (Lever et al., 2015). However, cell lysis should be avoided for iDNA and eDNA extraction. For example, the addition of low amounts of SDS has been reported to cause cell lysis for several methanogenic archaea (Wagner et al., 2013; Wagner, 2020; Wu & Lai, 2011), which would move DNA from the intracellular to the eDNA pool and create bias (Alawi et al., 2014). However, our data revealed no systematic indication that the DM lyses cells. Therefore, we assume that low amounts of Tween 80 and sodium pyrophosphate in the DM do not lead to cell lysis before separating cells from eDNA in the extraction procedure.

Although phosphate buffers are widely used in DNA extraction protocols (e.g. Alawi et al., 2014; Corinaldesi et al., 2005; Lever et al., 2015; Ogram et al., 1987), no study has yet reported on the relationship between phosphate concentrations and cell detachment processes. The implementation on the test sample from Pan de Azúcar (AZ‐2) revealed that the sole use of NaP 300 mM (without DM) did not improve iDNA recovery. This means that high phosphate concentrations in the extraction buffer do not increase cell detachment in our study.

Pure EDTA 300 mM buffer without DM showed a strong decrease of iDNA recovery for the low‐biomass sample from Pan de Azúcar (AZ‐2, Figure 2). This indicates that EDTA 300 mM alone is not appropriate to recover cells from inorganic, low‐biomass samples (e.g., AZ‐2) that contain, for instance, notable amounts of metal ions (Bernhard et al., 2018). However, the extraction of iDNA improved substantially after applying the combination of the phosphate buffer and EDTA 300 mM. Our result suggests that EDTA can have a beneficial effect on cell detachment when it is used together with a phosphate buffer. Little knowledge exists thus far on the effect of EDTA on cell detachment processes, and the role of EDTA in this process cannot yet be fully assessed. For example, certain organisms can attach to the mineral surfaces of iron oxides (Neal et al., 2005). The dissolution of iron oxides by EDTA (Nowack and Sigg, 1997) could lead to the release of cells and could, therefore, improve iDNA recovery. Additionally, one of the main components of biofilms is represented by extracellular polymeric substances (EPSs). These EPSs contain divalent cations (e.g., Mg^+2^ and Ca^+2^), which are essential to the integrity of the biofilm (Nielsen & Jahn, 1999). EDTA can detach cells from the matrix because it destabilizes the EPS by chelating divalent cations (Nielsen & Jahn, 1999), consequently increasing iDNA recovery.

Our alternative extraction setups successfully improved cell detachment as indicated by a correlation between increasing iDNA yields and cell counts for the modified methods on AZ‐2. While it is often difficult to compare DNA yields to direct cell counts, both measurements show similar improvement ratios with the updated methods. Furthermore, the spiked experiments showed no traces of added eDNA in the iDNA pool. This confirms that there is no cross‐contamination of eDNA on the filter membrane during the separation step. Particularly, in the spiked experiment using AZ‐2 with the NaP 300 mM + DM setup, the eDNA yield did not show the expected increase since the ratio was below < 1, we attributed this due to the effect of the DM which was involved in the reduction of eDNA yields. However, iDNA yields also decreased after adding genomic DNA by 78%. This reduction might be explained because of sample variability between the two extractions since 16 S rRNA results tend to vary more between different technical replicates, especially for low biomass samples like AZ‐2 (Claassen‐Weitz et al., 2020).

Summarizing the results of the iDNA yields of AZ‐2, the combination of highly concentrated phosphate buffer in combination with either DM or EDTA could improve cell detachment and, thus, increase iDNA yields.

### 4.3 DNA desorption: eDNA yields

In soils and sediments, eDNA is often adsorbed to particles by binding to ‐OH groups of for example iron oxides like ferrihydrite, negatively charged surfaces such as clay minerals, or organic matter (Saeki & Kunito, 2010). It is more challenging to extract when the eDNA is attached to mineral and soil particles. Therefore, maximizing the desorption of DNA molecules from mineral surfaces and organic compounds is critical to extract eDNA efficiently. The NaP 300 mM buffer without DM (NaP 300 mM) was the best extraction buffer for the AZ‐2 test sample. Large amounts of phosphate in extraction buffers are known to improve total DNA yields because phosphate competes with the negatively charged phosphate backbone of the DNA molecule for binding sites at charged mineral surfaces (Ogram et al., 1987). However, since the DNA adsorption capacity of the sample material is limited, an excess of phosphate due to highly concentrated buffers could be carried over and then compete with the ‐OH groups of the DNA for the silica membrane in the elution step, reducing the DNA yield (Lever et al., 2015; Lloyd et al., 2010). In addition, an excess of phosphate has also been suggested to lead to the co‐extraction of organic compounds (e.g., humic acids), which could also affect DNA recovery and inhibit DNA‐based analyses, such as PCR (Lever et al., 2015). Overall, phosphate concentration is an important aspect to consider in terms of the adsorption capacity of the sample, which can only be determined empirically (Lever et al., 2015). However, our data shows no indication of decreased eDNA recovery when using a higher concentrated sodium phosphate buffer exclusively (without DM).

High buffer concentrations often require sample incubation at high temperatures of over 60°C to prevent the precipitation of salts during DNA extraction. Incubations at high temperatures and any other major temperature fluctuations must be avoided in the iDNA and eDNA extraction protocols to ensure cell integrity during the washing step. Therefore, we kept our samples on ice during incubation. Although minor visible salt precipitation occasionally occurred while extracting with NaP 300 mM, iDNA and eDNA recovery were not negatively affected when using this setup, making 300 mM of sodium phosphate in the buffer suitable for extracting iDNA and eDNA. This aligns with Lever et al. (2015), who suggested using 100–1,000 μmol of PO_4_ g^–1^ soil for organic‐rich sediment samples and even higher concentrations for organic‐poor clay sediments due to their high sorption capacity. These values are comparable to the concentrations used in the present work, which were ~320 μmol of PO_4_ g^–1^ soil in the NaP 120 mM buffer and ~800 μmol of PO_4_ g^–1^ soil in the NaP 300 mM buffer.

The low eDNA yields when applying the EDTA 300 mM buffer to AZ‐2 indicate a negative effect of EDTA 300 mM on eDNA recovery. However, a concentration of 300 mM of EDTA has previously been used to improve total DNA desorption from volcanic ash soils that contained amorphous aluminum (Rai et al., 2010). The negative effect we observed with EDTA might be explained by the specific composition of our samples or by the possible co‐extraction of inhibitors. Consequently, when EDTA and sodium phosphate were used in the NaP+EDTA 300‐mM buffer, we observed a substantial decrease in eDNA recovery compared to NaP 300 mM alone. Thus, phosphate buffers are the most efficient agents for desorbing eDNA from the sample matrix.

Moreover, the precipitation of salts increased significantly after adding the DM to the sample buffer mixture because the DM contained 100 mM of sodium pyrophosphate. Precipitation during the washing step could explain the decrease of iDNA and eDNA yields after adding DM in some setups. Notably, such precipitation and its negative effect on DNA yields occur only occasionally and are therefore likely dependent on the specific sample composition (Lever et al., 2015).

### 4.4 Evaluation and method validation with different soil samples

**Figure A3:**
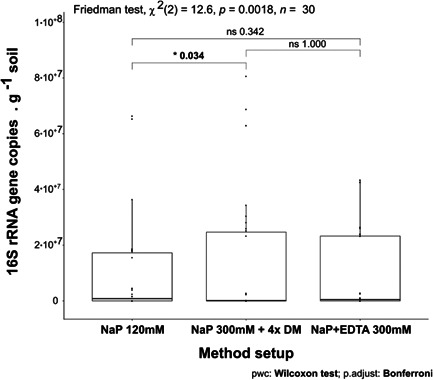
Friedman test and pairwise comparisons using paired Wilcoxon signed‐rank test on all iDNA samples with all extraction methods. *p* Values are adjusted using the Bonferroni multiple‐testing correction method. *p* < 0.05 were considered significant.

**Figure A4:**
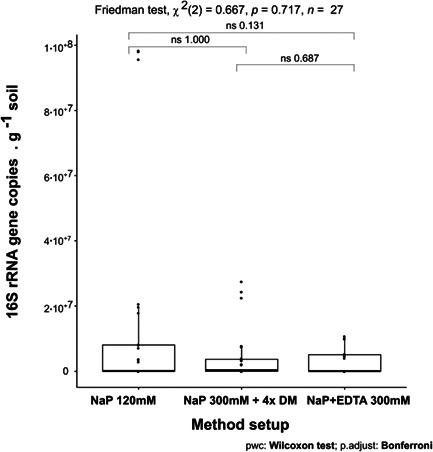
Friedman test and pairwise comparisons using paired Wilcoxon signed‐rank test on all eDNA samples with all extraction methods. *p* Values are adjusted using the Bonferroni multiple‐testing correction method. *p* < 0.05 were considered significant.

Based on the tests on the low biomass sample from the subsurface of Pan de Azúcar (AZ‐2) we identified an alternative protocol that significantly improved iDNA recovery as reflected in the increase of gene copy numbers g^–1^ and cell g^‐1^ soil while still providing sufficient eDNA yield for further downstream analyses. We tested the most promising NaP 300 mM buffer plus DM on various terrestrial samples that represented environments with different climate conditions and that also covered both surface and subsurface soils. With this setup, it was possible to increase iDNA for most of the analyzed samples (Friedman test *p* < 0.05, Appendix Figure A3).

Extreme environments often exhibit rare geochemical compositions, including high concentrations of metals or salts. Since the use of EDTA and phosphate buffer was successfully applied during DNA extractions from particular samples containing amorphous aluminum (Rai et al., 2010) and improved the iDNA recovery on the test sample AZ‐2, we also tested the NaP + EDTA 300 mM setup without DM on different terrestrial samples. This setup was applied to the same set of environmental samples as the NaP 300 mM buffer with the DM. The NaP+EDTA 300 mM setup was not statistically significant in comparison to the NaP 120 mM setup (Friedman test *p* = 0.342, Appendix Figure A3), however, it achieved improved iDNA recovery for seven samples. Notably, the successful extraction of iDNA from the highly challenging deep subsurface rock samples from Santa Gracia (SG‐34 and SG‐59) indicates that the amount of iDNA recovered with this method strongly depends on the geochemistry of the sample. For igneous rock samples containing iron minerals and weathering‐induced iron oxides, adding EDTA 300 mM as a chelating agent can be beneficial for iDNA extraction. On the other hand, for most of the samples, the best eDNA results were archived using pure phosphate buffers without additives (NaP 120 or 300 mM) because the addition of EDTA 300 mM and DM resulted in decreased eDNA concentrations. Nevertheless, the best eDNA result for deep subsurface samples SG‐34 and SG‐59 was achieved using the NaP 300‐mM + DM setup, again indicating the DM variable effect.

Our study aimed to improve the iDNA recovery using the iDNA and eDNA method developed by Alawi et al. (2014), focusing on samples from terrestrial environments, particularly low‐biomass surface and subsurface (deep biosphere) environments. We tested different buffers with and without DM during the washing step of the protocol. The alternative setups improved either cell detachment (iDNA yield) or DNA desorption (eDNA yield). Our study clearly shows no improvement for both DNA pools with the extraction setup. Instead, the protocol must be carefully selected according to the focus of the study. Therefore, for studies focusing on the living and potentially active part of the microbial community, represented by the iDNA pool, we recommend using the setups of NaP 300 mM + DM. For low‐biomass samples that contain high concentrations of metals or other ions, using the NaP + EDTA 300 mM setup may be beneficial to increase iDNA yields, as shown on the deep biosphere samples. If the goal is to investigate relic DNA as a potential record of past communities, the phosphate buffers of NaP 120 mM or 300 mM (without DM) are recommended since these setups were the most efficient in our eDNA extraction. Further studies should focus on specific soil properties to help identify the most appropriate extraction setup for the specific set of samples.

## AUTHOR CONTRIBUTIONS

**Diego Medina**: Conceptualization‐equal, data curation‐equal, formal analysis‐equal, investigation‐equal, methodology‐equal, validation‐equal, visualization‐equal, writing—original draft‐equal. **Lucas Horstmann**: Conceptualization‐equal, data curation‐equal, formal analysis‐equal, investigation‐equal, methodology‐equal, validation‐equal, visualization‐equal, writing—original draft‐equal. **Lars Ganzert**: Data curation‐supporting, methodology‐supporting, writing—review and editing‐supporting. **Romulo Oses**: Conceptualization‐supporting, funding acquisition‐equal, project administration‐equal, supervision‐equal, validation‐equal, writing—review and editing‐equal. **Thomas Friedl**: Conceptualization‐supporting, funding acquisition‐equal, project administration‐equal, resources‐equal, supervision‐equal, validation‐equal, writing—review and editing‐equal. **Dirk Wagner**: Conceptualization‐supporting, funding acquisition‐equal, project administration‐equal, resources‐equal, supervision‐lead, validation‐equal, writing—review and editing‐lead.

## CONFLICT OF INTEREST STATEMENT

None declared.

## ETHICS STATEMENT

None required.

## Data Availability

The datasets generated and analyzed in this study are available in the GFZ Data Services repository at https://doi.org/10.5880/GFZ.3.7.2023.001.

## 1

**Table A1:** iDNA yields from Pan de Azúcar subsurface sample (AZ‐2) using sodium phosphate buffer 120 mM (NaP 120 mM), sodium phosphate buffer 300 mM (NaP 300 mM), EDTA buffer 300 mM (EDTA 300 mM) or sodium phosphate‐EDTA buffer 300 mM (NaP + EDTA 300 mM) extraction setups.

**Buffer**	**Detergent mix addition**	**Replicate**	**16 S rRNA gene copy number (Gene copies g‐1)**
NaP 120 mM	No DM	1	8067.22
		2	6952.45
		3	7429.02
	DM	1	11,088.20
		2	12,432.90
		3	12,993.30
NaP 300 mM	No DM	1	7569.63
		2	5874.29
		3	7142.73
	DM	1	50,707.77
		2	53,060.93
		3	57,378.73
EDTA 300 mM	No DM	1	20,870.78
		2	22,892.80
		3	23,436.34
	DM	1	36,089.63
		2	38,619.63
		3	38,473.74
NaP/EDTA 300 mM	No DM	1	8067.22
		2	6952.45
		3	7429.02
	DM	1	11,088.20
		2	12,432.90
		3	12,993.30

*Note*: The column “Detergent mix addition” is indicating that was applied during all separation steps (DM) or in none of the separation steps (No DM) of the iDNA and eDNA extraction protocol. Missing values due to measuring errors or outliers are indicated as NA.

**Table A2:** eDNA yields from Pan de Azúcar subsurface sample (AZ‐2) using sodium phosphate buffer 120 mM (NaP 120 mM), sodium phosphate buffer 300 mM (NaP 300 mM), EDTA buffer 300 mM (EDTA 300 mM) or sodium phosphate‐EDTA buffer 300 mM (NaP+EDTA 300 mM) extraction setups.

**Buffer**	**Detergent mix addition**	**Replicate**	**16 S rRNA gene copy number (Gene copies g‐1)**
NaP 120 mM	No DM	1	70,824.12
		2	73,738.37
		3	67,922.70
	DM	1	5139.57
		2	4711.73
		3	4756.68
NaP 300 mM	No DM	1	164,773.69
		2	158,452.79
		3	164,151.83
	DM	1	47,206.12
		2	48,486.75
		3	50,246.48
EDTA 300 mM	No DM	1	3081.25
		2	2858.47
		3	3423.13
	DM	1	2063.56
		2	1894.79
		3	1818.48
NaP/EDTA 300 mM	No DM	1	70,824.12
		2	73,738.37
		3	67,922.70
	DM	1	5139.57
		2	4711.73
		3	4756.68

*Note*: The column “Detergent mix addition” is indicating that was applied during all separation steps (DM) or in none of the separation steps (No DM) of the iDNA and eDNA extraction protocol. Missing values due to measuring errors or outliers are indicated as NA.

**Table A3:** iDNA yields from all samples using the NaP 120 mM, NaP 300 mM +DM, and NaP + EDTA 300 mM setups.

**Extraction setup**	**Site**	**Sample**	**Replicate**	**DNA concentration (ng µL** ^**−1**^ **)**	**16 S rRNA gene copy number (Gene copies g** ^**−1**^ **)**	**Improvement in gene copy numbers**	**Improvement in DNA concentration**
NaP 120 mM	Nahuelbuta	NA‐1	1	2.78	66,349,832.96	NA	NA
			2	1.74	65,285,455.70	NA	NA
			3	1.15	36,412,971.61	NA	NA
	Nahuelbuta	NA‐2	1	1.28	18,685,144.53	NA	NA
			2	0.94	18,171,030.30	NA	NA
			3	1.25	NA	NA	NA
	Pan de Azúcar	AZ‐1	1	0.01	4662.07	NA	NA
			2	0.01	5249.04	NA	NA
			3	0.01	7597.52	NA	NA
	Pan de Azúcar	AZ‐2	1	0.01	8067.22	NA	NA
			2	0.01	6952.45	NA	NA
			3	0.01	7429.02	NA	NA
	La Campana	LC‐1	1	0.41	3,918,250.71	NA	NA
			2	0.25	4,428,102.38	NA	NA
			3	0.25	4,573,697.76	NA	NA
	La Campana	LC‐2	1	0.41	43,723.93	NA	NA
			2	0.25	65,604.70	NA	NA
			3	0.25	49,891.83	NA	NA
	Santa Gracia	SG‐1	1	1.31	18,076,989.52	NA	NA
			2	1.35	17,826,451.28	NA	NA
			3	1.40	15,572,199.70	NA	NA
	Santa Gracia	SG‐2	1	0.08	1,608,929.25	NA	NA
			2	0.07	2,426,533.67	NA	NA
			3	0.10	1,608,366.61	NA	NA
	Deep Biosphere	SG‐34	1	0.01	396.00	NA	NA
			2	0.01	396.00	NA	NA
			3	0.01	396.00	NA	NA
	Deep Biosphere	SG‐59	1	0.01	396.00	NA	NA
			2	0.01	396.00	NA	NA
			3	0.01	396.00	NA	NA
NaP 300 mM + DM	Nahuelbuta	NA‐1	1	12.60	80,599,121.16	1.21	4.53
			2	10.10	68,769,142.08	1.05	5.80
			3	7.55	62,908,368.46	1.73	6.57
	Nahuelbuta	NA‐2	1	5.55	23,312,773.81	1.25	4.34
			2	4.28	25,188,630.56	1.39	4.55
			3	5.58	26,002,461.26	NA	4.46
	Pan de Azúcar	AZ‐1	1	0.02	139,951.87	30.02	2.20
			2	0.02	116,415.36	22.18	1.70
			3	0.02	127,240.19	16.75	1.90
	Pan de Azúcar	AZ‐2	1	0.01	50,707.77	6.29	1.00
			2	0.01	53,060.93	7.63	1.00
			3	0.01	57,378.73	7.72	1.00
	La Campana	LC‐1	1	2.30	2,779,735.76	0.71	5.61
			2	1.57	2,514,179.68	0.57	6.33
			3	1.12	2,280,962.57	0.50	4.41
	La Campana	LC‐2	1	0.03	73,531.70	1.68	0.07
			2	0.02	73,283.53	1.12	0.10
			3	0.01	95,678.75	1.92	0.06
	Santa Gracia	SG‐1	1	4.09	34,370,706.87	1.90	3.12
			2	4.26	30,448,527.77	1.71	3.16
			3	3.94	28148965.55	1.81	2.81
	Santa Gracia	SG‐2	1	0.06	167,470.43	0.10	0.70
			2	0.02	185,025.35	0.08	0.24
			3	0.02	142,326.10	0.09	0.20
NaP 300 mM + D	Deep Biosphere	SG‐34	1	0.01	1350.35	3.41	1.00
			2	0.01	1431.79	3.62	1.00
			3	0.01	1495.34	3.78	1.00
	Deep Biosphere	SG‐59	1	NA	NA	NA	NA
			2	NA	NA	NA	NA
			3	NA	NA	NA	NA
	Nahuelbuta	NA‐1	1	0.78	2,568,300.02	0.04	0.28
			2	0.76	2,814,921.62	0.04	0.43
			3	0.75	2,644,195.41	0.07	0.65
	Nahuelbuta	NA‐2	1	7.58	42,565,585.78	2.28	5.92
			2	7.79	4,3362,470.69	2.39	8.29
			3	7.43	42,578,636.11	NA	5.94
NaP + EDTA 300 mM	Pan de Azúcar	AZ‐1	1	0.01	24,160.08	5.18	1.00
			2	0.01	22,286.58	4.25	1.00
			3	0.01	19,781.02	2.60	1.00
	Pan de Azúcar	AZ‐2	1	0.01	20,870.78	2.59	1.00
			2	0.01	22,892.80	3.29	1.00
			3	0.01	23,436.34	3.15	1.00
	La Campana	LC‐1	1	3.76	26,086,614.36	6.66	9.17
			2	4.86	26,485,189.02	5.98	19.60
			3	2.81	23,298,089.18	5.09	11.06
	La Campana	LC‐2	1	0.01	60,198.67	1.38	0.02
			2	0.01	50,687.32	0.77	0.04
			3	0.01	57,105.12	1.14	0.04
	Santa Gracia	SG‐1	1	3.40	23,206,139.86	1.28	2.60
			2	2.82	24,022,124.22	1.35	2.09
			3	2.96	23,886,960.75	1.53	2.11
	Santa Gracia	SG‐2	1	0.10	924,711.43	0.57	1.26
			2	0.11	1,101,594.88	0.45	1.70
			3	0.08	1,000,316.92	0.62	0.77
	Deep biosphere	SG‐34	1	0.03	2035.93	5.14	3.10
			2	0.01	2141.40	5.41	1.00
			3	0.04	2151.42	5.43	3.80
	Deep biosphere	SG‐59	1	0.01	2230.30	5.63	1.00
			2	0.01	1822.98	4.60	1.00
			3	0.01	1963.46	4.96	1.00

*Note*: Missing values due to measuring errors or outliers are indicated as NA. As the improvement factors are based on the relative improvement compared to the NaP 120 mM, improvement factors for this setup are indicated as NA. For calculation and visualization of improvements for samples that were not measurable with the NaP 120 mM method, the detection limit of the Qubit fluorometer or the respective qPCR run was used. These values are marked in italicin this table. Detergent‐mix (DM).

**Table A4:** eDNA yields from all samples using the NaP 120 mM, NaP 300 mM +DM, and NaP+EDTA 300 mM setups.

**Extraction setup**	**Site**	**Sample**	**Replicate**	**DNA concentration (ng µL** ^**−1**^ **)**	**16S rRNA gene copy number (Gene copies g** ^**−1**^ **)**	**Improvement in gene copy numbers**	**Improvement in DNA concentration**
NaP 120 mM	Nahuelbuta	NA‐1	1	68.90	9,983,479.24	NA	NA
			2	119.00	10,241,132.22	NA	NA
			3	102.00	8,897,406.40	NA	NA
	Nahuelbuta	NA‐2	1	52.00	4,971,875.26	NA	NA
			2	55.00	4,642,306.34	NA	NA
			3	52.00	4,665,952.04	NA	NA
	Pan de Azúcar	AZ‐1	1	0.01	8952.46	NA	NA
			2	0.01	7215.97	NA	NA
			3	0.01	11,380.55	NA	NA
	Pan de Azúcar	AZ‐2	1	0.01	70,824.12	NA	NA
			2	0.01	73,738.37	NA	NA
			3	0.02	67,922.70	NA	NA
	La Campana	LC‐1	1	4.64	12,2540,111.00	NA	NA
			2	3.42	122,910,779.80	NA	NA
			3	5.72	119,549,815.70	NA	NA
	La Campana	LC‐2	1	0.05	183,423.11	NA	NA
			2	0.05	177,623.57	NA	NA
			3	0.05	175,764.83	NA	NA
	Santa Gracia	SG−1	1	0.44	4,647,195.52	NA	NA
			2	0.32	3,630,754.97	NA	NA
			3	0.36	4,428,639.64	NA	NA
	Santa Gracia	SG‐2	1	3.28	22,364,652.58	NA	NA
			2	2.62	24,654,443.51	NA	NA
			3	3.22	25,739,458.51	NA	NA
	Deep biosphere	SG‐34	1	0.01	*7400*	NA	NA
			2	0.01	*7400*	NA	NA
			3	0.01	*7400*	NA	NA
	Deep biosphere	SG‐59	1	0.01	*7400*	NA	NA
			2	0.01	*7400*	NA	NA
			3	0.01	*7400*	NA	NA
NaP 300 mM + DM	Nahuelbuta	NA‐1	1	0.28	4,305,063.78	0.43	0.00
			2	0.35	4,908,358.53	0.48	0.00
			3	0.32	4,048,106.68	0.45	0.00
	Nahuelbuta	NA‐2	1	0.35	31,184,284,568.00	6272.14	0.01
			2	0.57	29,845,883,061.00	6429.11	0.01
			3	0.36	29,114,678,382.00	6239.82	0.01
	Pan de Azúcar	AZ‐1	1	0.10	442,846.46	49.47	10.40
			2	0.13	471,772.74	65.38	13.00
			3	0.05	456,167.28	40.08	4.60
	Pan de Azúcar	AZ‐2	1	0.01	47,206.12	0.67	1.20
			2	0.02	48,486.75	0.66	1.70
			3	0.01	50,246.48	0.74	0.41
	La Campana	LC‐1	1	0.25	2,485,401.57	0.02	0.05
			2	0.26	2,706,972.95	0.02	0.08
			3	0.26	2,685,305.87	0.02	0.04
	La Campana	LC‐2	1	0.03	208,654.10	1.14	0.68
			2	0.06	192,733.91	1.09	1.07
			3	0.04	211,975.79	1.21	0.83
	Santa Gracia	SG‐1	1	1.05	NA	NA	2.36
			2	1.09	NA	NA	3.39
			3	0.88	NA	NA	2.42
	Santa Gracia	SG‐2	1	1.95	9,455,520.89	0.42	0.59
			2	1.68	9,314,070.82	0.38	0.64
			3	1.43	9,738,871.07	0.38	0.44
	Deep biosphere	SG‐34	1	0.01	1643.69	22.21	1.00
			2	0.01	1424.96	19.26	1.00
			3	0.01	1720.33	23.25	1.00
	Deep biosphere	SG‐59	1	0.01	1542.14	20.84	1.00
			2	0.01	1509.40	20.40	1.00
			3	0.01	1655.22	22.37	1.00
NaP + EDTA 300 mM	Nahuelbuta	NA‐1	1	1.73	5,771,383.46	0.58	0.03
			2	1.56	5,696,287.83	0.56	0.01
			3	0.99	6,266,577.73	0.70	0.01
	Nahuelbuta	NA‐2	1	0.01	2,071,572.81	0.42	0.00
			2	0.01	2,528,593.76	0.54	0.00
			3	0.01	2,162,055.30	0.46	0.00
	Pan de Azúcar	AZ‐1	1	0.32	32,818.48	3.67	31.80
			2	0.27	36,234.42	5.02	27.40
			3	0.46	45,759.70	4.02	45.90
	Pan de Azúcar	AZ‐2	1	0.01	3081.25	0.05	1.00
			2	0.01	2858.47	0.04	1.00
			3	0.01	3423.13	0.04	0.59
	La Campana	LC−1	1	0.94	6,070,565.88	0.05	0.20
			2	0.92	6,662,944.22	0.05	0.27
			3	0.90	5,006,338.95	0.04	0.16
	La Campana	LC‐2	1	0.05	130,938.95	0.71	1.12
			2	0.03	91,460.16	0.51	0.56
			3	0.03	97,775.02	0.56	0.49
	Santa Gracia	SG‐1	1	0.21	13,456,232.00	2.90	0.48
			2	0.41	12,394,857.07	3.41	1.27
			3	0.27	12,708,002.79	2.87	0.73
	Santa Gracia	SG‐2	1	0.01	6,604,134.77	0.30	0.00
			2	0.01	6,511,865.06	0.26	0.00
			3	0.01	6,541,434.77	0.25	0.00
	Deep biosphere	SG‐34	1	NA	NA	NA	NA
			2	NA	NA	NA	NA
			3	NA	NA	NA	NA
	Deep biosphere	SG‐59	1	NA	NA	NA	NA
			2	NA	NA	NA	NA
			3	NA	NA	NA	NA

*Note*: Missing values due to measuring errors or outliers are indicated as NA. As the improvement factors are based on the relative improvement compared to the NaP 120 mM, improvement factors for this setup are indicated as NA. For calculation and visualization of improvements for samples that were not measurable with the NaP 120 mM method, the detection limit of the Qubit fluorometer or the respective qPCR run was used. These values are marked in italicin this table. Detergent‐mix (DM).

**Table A5:** Cell counting of the test sample AZ‐2 using the “NaP 120 mM”. “NaP 300 mM + DM” and “NaP+EDTA 300 mM” setups.

**Setup**	**Sample weight (g)**	**FOV**	**Total cells counted**	**Dilution factor**	**Cell number (cells g** ^**−1**^ **)**	**Average cell number (cells g** ^**−1**^ **)**
NaP 120 mM	3.000	200	42	3	4157.13	2.97 × 10^3^
	3.000	200	18	3	1781.63	
	3.000	200	30	3	2969.38	
NaP 300 mM + DM	3.000	200	155	3	15,281.61	1.08 × 10^4^
	3.000	200	40	3	3943.64	
	3.000	200	135	3	13,309.79	
NaP + EDTA 300 mM	3.000	200	36	3	3562.09	2.82 × 10^4^
	3.000	200	180	3	17,810.43	
	3.000	63	201	3	63,137.51	

**Table A6:** Spiked experiment with the Buffer NaP 120 mM and sand.

**Sample**	**Replicate**	**eDNA or iDNA**—**nonspiked**	**eDNA or iDNA** + **500 ug genomic DNA**—**spiked**	**Ratio spiked/nonspiked**	**Average ratio**
NaP 120 mM—eDNA	1	17.73	2645.12	149.19	189.17
	2	14.56	3025.26	207.85	
	3	15.97	3361.47	210.46	
Sand—eDNA	1	15.59	6807.59	436.64	481.93
	2	14.64	7544.90	515.48	
	3	14.75	7280.85	493.69	
NaP 120 mM—iDNA	1	30.31	44.02	1.45	1.49
	2	28.67	36.00	1.26	
	3	24.62	43.47	1.77	
Sand—iDNA	1	29.03	70.15	2.42	2.13
	2	33.06	51.90	1.57	
	3	32.69	78.43	2.40	

*Note*: eDNA and iDNA yields, with raw qPCR data, of nonspiked and 1 μg DNA spiked with the Buffer NaP 120 mM and free‐DNA combusted sand.

**Table A7:** Spiked experiment with AZ‐2.

**Setup**	**Replicate**	**sample weight (g)**	**eDNA or iDNA nonspiked**	**eDNA or iDNA + 500 ug genomic DNA—Spiked**	**Ratio Spiked/nonspiked**	**Average ratio**
NaP 120 mM**—**eDNA	**1**	3.000	70,824.12	713,626.57	10.08	8.41
	**2**	3.000	73,738.37	527,318.68	7.15	
	**3**	3.000	67,922.70	544,191.55	8.01	
NaP 300 mM + DM**—**eDNA	**1**	3.000	47,206.12	27,561.60	0.58	0.55
	**2**	3.000	48,486.75	25,821.57	0.53	
	**3**	3.000	50,246.48	26,758.40	0.53	
NaP/EDTA 300 mM**—**eDNA	**1**	3.000	3081.25	23,203.66	7.53	7.15
	**2**	3.000	2858.47	20,580.61	7.20	
	**3**	3.000	3423.13	22,967.75	6.71	
NaP 120 mM ‐ iDNA	**1**	3.000	8067.22	6485.90	0.80	0.85
	**2**	3.000	6952.45	6510.83	0.94	
	**3**	3.000	7429.02	6080.38	0.82	
NaP 300 mM + DM—iDNA	**1**	3.000	50,707.77	9636.67	0.19	0.20
	**2**	3.000	53,060.93	11,255.95	0.21	
	**3**	3.000	57,378.73	10,493.04	0.18	
NaP/EDTA 300 mM**—**iDNA	**1**	3.000	20,870.78	22,612.75	1.08	1.04
	**2**	3.000	22,892.80	23,893.23	1.04	
	**3**	3.000	23,436.34	23,403.59	1.00	

*Note*: eDNA and iDNA yields, yields, in 16 S gene copy number g‐1, of the non‐spiked and 500 ng genomic DNA spiked with the test sample AZ‐2 using “NaP 120 mM”. “NaP 300 mM + DM” and “NaP+EDTA 300 mM” setups.
